# The intersection of the two complimentary fields of preventive cardiology and integrative cardiology: A Fellow's voice

**DOI:** 10.1016/j.ajpc.2022.100433

**Published:** 2022-11-19

**Authors:** Brian Cheung

**Affiliations:** a Susan Samueli Integrative Health Institute, University of California, Irvine, CA, United States; b Division of Cardiology, University of California, Irvine, CA, United States

**Keywords:** Acupuncture, Alternative medicine, Biomarkers, Biofeedback, Cardiovascular training, Complementary medicine, Diet, Exercise, Holistic health, Integrative Medicine, Massage therapy, Meditation, Mindful eating, Mindfulness-based stress reduction, Nutrition, Osteopathic manipulative therapy, Qigong, Tai Chi, Wellness, Yoga, SSIHI, Susan Samueli Integrative Health Institute

During my internal medicine residency, I worked closely with one of my patients who had a history of obesity and hypertension. Despite being a busy single mother of 3 in a rigorous Doctor of Philosophy program, she decided to take charge of her life and was motivated to make drastic lifestyle changes. She cut out a lot of processed foods and practiced mindful eating. She began a daily workout routine, started yoga, and practiced mindful meditation every morning when she had her coffee. This helped with stress reduction as she was able to acknowledge her busy work life and to clear her mind. She was able to lose weight, to reduce her blood pressure, and to discontinue her lisinopril.

I will always remember her sense of pride and accomplishment that she was able to achieve. Through her holistic approach to health and wellness, she taught me so much about prevention and integrative medicine. She sowed these seeds, which eventually germinated and blossomed with me formally pursuing these passions so that I could better advise and care for my patients from a more holistic approach.

I have had the unique honor and privilege of being the former Katz Foundation Preventive Cardiology Post-Doctoral Fellow at Emory University and the current Susan Samueli Integrative Health Institute (SSIHI) Integrative Cardiology Fellow at the University of California, Irvine. These fellowship programs have been made possible through the generous donations of philanthropists who are dedicated to cardiovascular prevention and to creating the next generation of academic leaders who will shape how cardiovascular care will be delivered tomorrow. Both programs are at premiere institutions with state-of-the-art facilities designed to train the future leaders in cardiovascular prevention through a multitude of modalities. Fellows have the opportunity to be mentored by world renowned National Institutes of Health funded physician-scientists and faculty who lead and founded national and international cardiology societies, write many society guidelines and statements, and are editors of many prestigious high-impact journals. Trainees get a plethora of rich clinical research experience coupled with clinical training in preventive and integrative cardiology.

Through the SSIHI integrative cardiology fellowship, I had many unique opportunities to learn more about this new and exciting field. Integrative cardiology incorporates aspects of both conventional and integrative medicine to provide care that is congruent with the most up-to-date body of knowledge and guidelines on prevention. It leverages current therapeutic modalities and lifestyle interventions to provide individual care that is tailored to their personal risk and aligns with their beliefs, values, and goals. It encompasses a multidimensional outlook of health and wellness that extends beyond symptom management and disease.

Integrative cardiology aims to provide care that addresses the whole person, which is essential and foundational for a modern healthcare system. Health is intrinsically holistic because it embodies many overlapping spheres in a patient's life, such as the intricate interplay between the physical, mental, emotional, spiritual, social, economic, and environmental arenas. Possible services provided can include culinary medicine courses, mindful eating, mindfulness-based stress reduction courses, weight management, exercise prescriptions, acupuncture, massage therapy, meditation, yoga, tai chi, qigong, biofeedback, coaching, osteopathic manipulative therapy, and more. To properly serve patients with this multitude of services, it requires a team-based and interdisciplinary approach. Depending on the needs of each individual patient, an integrative cardiology care team can include medical specialists, pharmacists, behavioral health professionals, naturopathic doctors, nutritionists, physical therapists, exercise physiologists, acupuncturists, massage therapists, mindfulness instructors, yoga therapists, health coaches, patient advocates, and more.

Integrative cardiology shares many commonalities with preventive cardiology in terms of philosophies, goals, and treatment modalities. Both subspecialties in cardiology aim to maximize the duration that patients spend in good health by promoting health, wellness, and prevention. Patients are invited to take an active role in the management of their health and to empower them to make investments in themselves that will yield dividends in their later years. Both subspecialties also focus on partnering with the community and to provide education about cardiac health in order to activate and to galvanize others to make healthy lifestyle choices.

Modalities traditionally thought of as integrative therapies are endorsed by leaders in preventive cardiology. In a *Journal of the American College of Cardiology* Council perspective, the use of natural therapies, nutrition, lifestyle modification, and stress management were specifically mentioned as necessary competencies for preventive cardiology [1]. The authors also highlighted the importance of developing competency in the management of diabetes, hypertension, dyslipidemia, obesity, and smoking cessation and underscored the use of biomarkers, genetics, cardiovascular imaging, and stress testing when risk stratifying patients [1]. These are all competencies that are also integral to the daily practice of integrative cardiology in order to provide care for the whole person in mind.

Many recent developments have led to the tipping point for the increasingly wide scale adoption of integrative medicine in the care of patients with cardiovascular disease. Meditation, yoga, and mindful eating are recommended in the guidelines, scientific statements, and editorials from both the American Heart Association and the American College of Cardiology in the management of stress, hypertension, and primary prevention of cardiovascular disease [2], [3], [4], [5]. Integrative therapies have seen mainstream adoption in the Department of Defense and the Department of Veteran Affairs, and acupuncture, massage therapy, meditation, yoga, tai chi, and qigong are offered to active service members, veterans, and their families [6]. The Bravewell Integrative Medicine Research Network (BraveNet), which is a practice-based research collaborative network of over 15+ premier integrative medicine centers in the United States, was established in 2007 to investigate and generate evidence-based research in integrative medicine [7].

There is an unmet need for dedicated specialized training in both preventive and integrative cardiology in order to train the leaders of tomorrow. Specialized clinical expertise is needed to effectively address cardiometabolic risk factors, such as dyslipidemia, hypertension, obesity, diabetes, and physical inactivity at the large societal scale. There is a growing demand for prevention that focuses on health and wellness. Specialists in preventive and integrative cardiology stand together at the forefront to champion these ideals for our patients.

## Statement of funding

This manuscript received no funding.

## CRediT authorship contribution statement

**Brian Cheung:** Conceptualization, Writing – original draft.

## Declaration of Competing Interest

The authors declare that they have no known competing financial interests or personal relationships that could have appeared to influence the work reported in this paper.
